# Development and validation of a new risk assessment model for immunomodulatory drug-associated venous thrombosis among Chinese patients with multiple myeloma

**DOI:** 10.1186/s12959-023-00534-y

**Published:** 2023-10-04

**Authors:** Xiaozhe Li, Xiuli Sun, Baijun Fang, Yun Leng, Fangfang Sun, Yaomei Wang, Qing Wang, Jie Jin, Min Yang, Bing Xu, Zhihong Fang, Lijuan Chen, Zhi Chen, Qimei Yang, Kejie Zhang, Yinhai Ye, Hui Geng, Zhiqiang Sun, Dan Hao, Hongming Huang, Xiaotao Wang, Hongmei Jing, Lan Ma, Xueyi Pan, Wenming Chen, Juan Li

**Affiliations:** 1 Department of Haematology, First Affiliated Hospital of Sun Yat-sen University, No. 58 Zhongshan Er Road, Guangzhou, 510000 Guangdong China; 2 Department of Haematology, First Affiliated Hospital of Dalian Medical University, Dalian, China; 3 Department of Haematology, Henan Cancer Hospital, Zhengzhou, China; 4 Department of Haematology, Beijing Chaoyang Hospital, Beijing, China; 5 Department of Haematology, Guizhou Provincial People’s Hospital, Guiyang, China; 6 Department of Haematology, First Affiliated Hospital of Zhejiang University School of Medicine, Hangzhou, China; 7 Department of Haematology, First Affiliated Hospital of Xiamen University, Xiamen, China; 8 Department of Haematology, Jiangsu Provincial People’s Hospital, Nanjing, China; 9 Department of Haematology, Shantou Central Hospital, Shantou, China; 10 Department of Haematology, Zhongshan Hospital Affiliated to Xiamen University, Xiamen, China; 11 Department of Haematology, Affiliated Hospital of Qinghai University, Xining, China; 12 Department of Haematology, Shenzhen Hospital of Southern Medical University, Shenzhen, China; 13 Department of Haematology, Nantong University Hospital, Nantong, China; 14 Department of Haematology, Second Affiliated Hospital of Guilin Medical College, Guilin, China; 15 Department of Haematology, Peking University Third Hospital, Beijing, China; 16 Department of Haematology, First Affiliated Hospital of Guangdong Pharmaceutical University, Guangzhou, China

**Keywords:** Immunomodulatory drugs, Multiple myeloma, Risk assessment model, Venous thromboembolism, Thromboprophylaxis

## Abstract

**Background:**

Individuals with multiple myeloma (MM) receiving immunomodulatory drugs (IMiDs) are at risk of developing venous thromboembolism (VTE), a serious complication. There is no established clinical model for predicting VTE in the Chinese population. We develop a new risk assessment model (RAM) for IMiD-associated VTE in Chinese MM patients.

**Methods:**

We retrospectively selected 1334 consecutive MM patients receiving IMiDs from 16 medical centers in China and classified them randomly into the derivation and validation cohorts. A multivariate Cox regression model was used for analysis.

**Results:**

The overall incidence of IMiD-related VTE in Chinese MM patients was 6.1%. Independent predictive factors of VTE (diabetes, ECOG performance status, erythropoietin-stimulating agent use, dexamethasone use, and VTE history or family history of thrombosis) were identified and merged to develop the RAM. The model identified approximately 30% of the patients in each cohort at high risk for VTE. The hazard ratios (HRs) were 6.08 (P < 0.001) and 6.23 (P < 0.001) for the high-risk subcohort and the low-risk subcohort, respectively, within both the derivation and validation cohorts. The RAM achieved satisfactory discrimination with a C statistic of 0.64. The stratification approach of the IMWG guidelines yielded respective HRs of 1.77 (P = 0.053) and 1.81 (P = 0.063). The stratification approach of the SAVED score resulted in HRs of 3.23 (P = 0.248) and 1.65 (P = 0.622), respectively. The IMWG guideline and the SAVED score-based method yielded C statistics of 0.58 and 0.51, respectively.

**Conclusions:**

The new RAM outperformed the IMWG guidelines and the SAVED score and could potentially guide the VTE prophylaxis strategy for Chinese MM patients.

**Supplementary Information:**

The online version contains supplementary material available at 10.1186/s12959-023-00534-y.

## Background

Venous thromboembolism (VTE) is correlated with cancer and is a significant cause of long-term morbidity, deteriorative quality of life, and negative psychological outcomes. Moreover, it represents a considerable financial and resource burden on healthcare systems and is correlated with increased mortality rates [1, 2]. Patients with multiple myeloma (MM) have a 9-fold greater risk of VTE than the general population, which makes it a particularly dangerous disease [3]. The death rate in MM patients with VTE is three times greater than in MM patients without VTE within one year [4].

Immunomodulatory drugs (IMiDs), such as lenalidomide and thalidomide, which improve the outcomes of MM, are key drugs in the current treatment of MM. Still, they pose a significantly higher risk of VTE events [5]. During the course of the disease, the incidence of VTE in MM patients is approximately 10%. Studies in Western countries indicate that IMiDs in MM therapy could increase the incidence of VTE, ranging from 14 to 75% [6–10]. The mechanisms that cause the elevated risk of VTE secondary to administering IMiDs for treating MM are possibly multifactorial, including treatment-related factors, patient‐specific factors, and specific pathophysiologic changes [4, 11, 12]. Hence, identifying the risk factors associated with VTE is crucial for preventing its occurrence in MM patients receiving IMiDs.

In 2014, the International Myeloma Working Group (IMWG) published guidelines for preventing VTE in MM patients based on various risk factors for VTE [13, 14]. These guidelines recommended antiplatelet therapy for patients with 0–1 risk factors and anticoagulation therapy for patients with more than 1 risk factor. Although experts developed this risk stratification guideline to assist clinicians in determining the appropriate patients for pharmacologic thromboprophylaxis, it has yet to be externally validated, and its predictive accuracy for VTE is suboptimal. The guideline of IMWG did not consider ethnic differences [6, 15, 16].

However, Asian-Pacific Islanders generally have a lower risk of developing VTE in contrast with the general population [17], and based on current literature reports, even Asian myeloma patients who use IMiDs have a relatively reduced risk of VTE. Anecdotal reports from Asia suggest that the incidence rate of VTE with IMiDs ranges from 2 to 8.3% [18–20]. The hypothesis that all patients should receive antiplatelet or anticoagulation therapy to prevent VTE, based on IMWG guidelines, may not effectively prevent thrombosis and may increase financial burden and bleeding risk. Therefore, the IMWG guidelines are unsuitable for predicting VTE in MM Chinese patients.

Several data-driven risk assessment models, such as IMPEDE-VTE and SAVED, have recently been developed and validated [15, 21]. During the initial anti-myeloma therapy, IMPEDE-VTE was aimed at all patients with MM. In contrast, the SAVED score is a 5-point model specifically developed for MM patients receiving IMiDs, and it has recently been included in the National Comprehensive Cancer Network (NCCN) guidelines [22]. The NCCN emphasized that this recommendation is based on lower-level evidence with a category of evidence 2 A, and most initial studies were conducted in Western countries. The proportion of Asian individuals in these studies was less than 10% of the sample size in the SAVED score. Hence, it remains uncertain whether the SAVED score applies to Asian populations [15, 21]. The present research aimed to investigate the incidence rate of VTE and the risk factors related to it in Chinese MM patients receiving IMiDs. We also conducted the largest multicentre study in China to develop and verify a novel risk assessment model (RAM) for IMiD-related VTE among Chinese MM patients.

## Methods

### Patients and study design

Between January 2010 and March 2020, we performed a retrospective multicenter cohort study of MM patients who received IMiDs at 16 leading academic medical centers in China. The study aimed to develop and validate a multivariate predictive model. We conducted a retrospective study to collect demographic and clinical data of patients who received IMiD-based treatment for at least 7 days. The incidence of VTE events was the primary outcome measure. Patients with incomplete data for risk score calculation, receiving therapeutic anticoagulation at the time of IMiD initiation or diagnosed with VTE within 30 days before the first IMiD prescription were excluded from the study. Patients who died from any cause before VTE within 12 months of treatment were not excluded from the analysis.

**Fig. 1:**
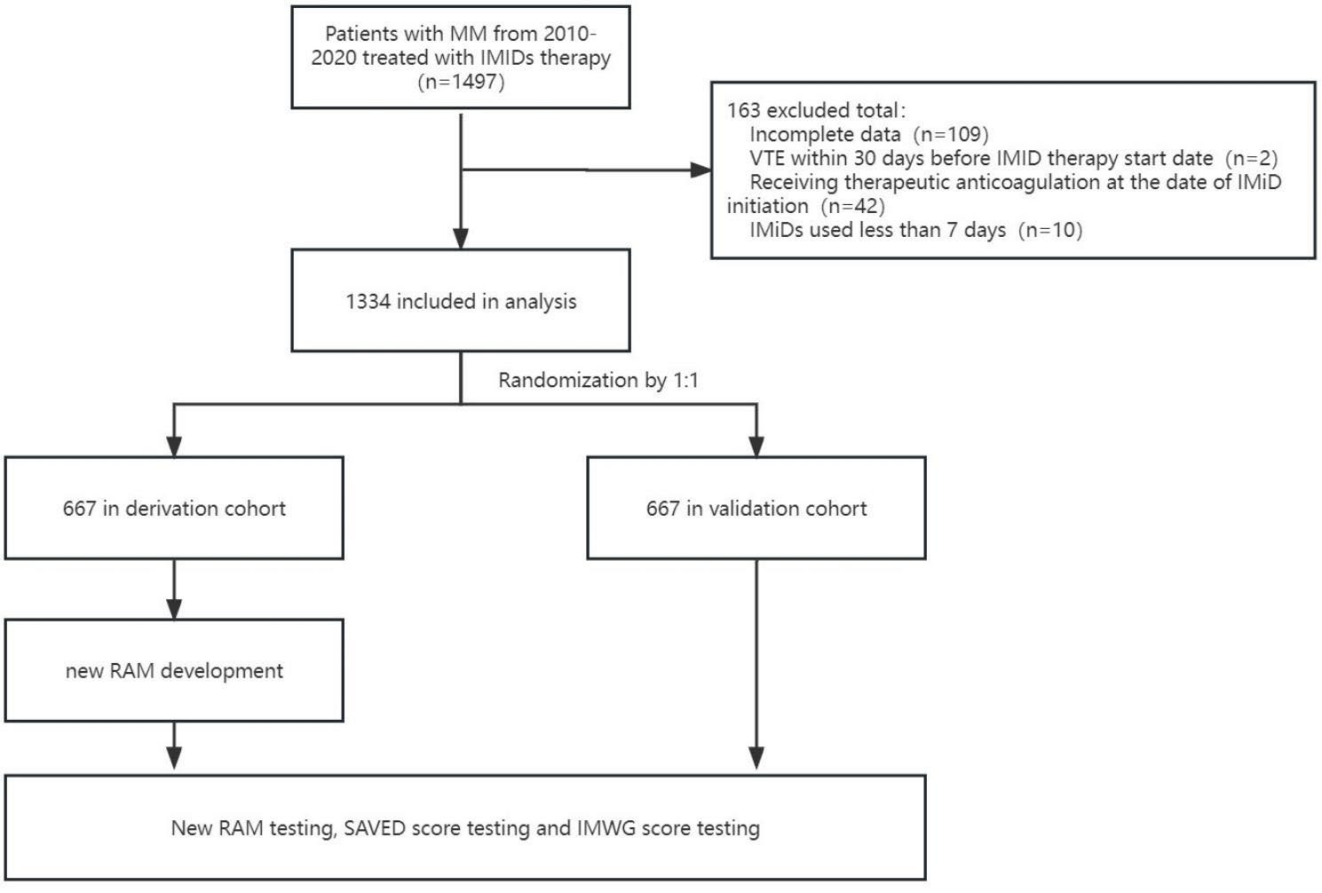
Patient enrolment and model development

The enrolled patients were randomly assigned to the derivation and validation cohorts for subsequent analysis. In this study, randomization was accomplished by assigning a number to each patient using a computer-generated sequence and then randomly allocating them to the two groups according to their assigned number at a 1:1 ratio. Eventually, 667 patients were assigned to the derivation cohort, and 667 patients were enrolled in the validation cohort (Fig. 1). Each hospital’s institutional review board examined and approved the research protocol before it could be implemented. The requirements of the *Declaration of Helsinki* for biomedical research involving human participants were adhered to.

### Variables and outcome

All the patients who developed VTE were symptomatic; their diagnosis was confirmed with conventional angiography and/or angiography utilizing magnetic resonance imaging or computed tomography. VTE was defined as developing a pulmonary embolism or any VTE of the lower or upper extremities, but we excluded superficial venous thromboses. The patients were subjected to follow-up from the time of IMiD treatment initiation until the onset of VTE, death, the first day following discontinuation of IMiD treatment, the time of loss to follow-up, or until March 2020 (whichever occurred first). Patients were followed up within one year of IMID initiation for any VTE episode to establish the VTE risk prediction model. The potential risk factors for VTE, including individual factors, disease information, treatment factors, and laboratory parameters, are outlined in Supplementary Tables 1 and described below.

The risk factors for venous thrombosis were previously identified with the aid of the IMWG guidelines and included central vein access device or pacemaker, prior VTE, obesity (body mass index (BMI) ≥ 28 kg/m2), related diseases (immobilization, acute infection, diabetes, chronic renal disease, and cardiac disease), surgical operation (trauma, any anaesthesia, and general surgery), erythropoietin use, high-dose dexamethasone (≥ 480 mg/month), and multiagent chemotherapy and/or doxorubicin treatment. We assessed the height and weight of the patients starting 30 days before the beginning of IMiD treatment. BMI was derived for the patients utilizing their weight and height measurements. We successfully obtained the estimated glomerular filtration rate (eGFR) using the Chronic Kidney Disease Epidemiology Collaboration algorithm. The dexamethasone dosage for each cycle was computed from the mean monthly dexamethasone dosage. We defined recent surgery as any surgical procedure performed within 30 days before initiating IMiD therapy. We defined patients with a fracture as those who had experienced a fracture within 30 days before the initiation of IMiD treatment. To validate the SAVED score, we calculated the SAVED model at IMiD initiation according to the following five parameters: surgery within previous 90 days (+ 2 points) defined as major thoracic, neurologic, orthopaedic, abdominal, or urological procedure; Asian race (− 3 points); prior VTE history (+ 3 points); age 80 years or above (+ 1 point); and dexamethasone dose over the 30-­day course (+ 1 point for standard and + 2 points for high dose). A standard dose of dexamethasone was defined as 120–160 mg/month, and a high dose was > 160 mg/month. For surgery history, general surgery or any anesthesia was considered. Patients who scored ≥ 2 points were classified as high risk, while those with a score ≤ 1 were classified as low risk [15].

To identify any additional risk factors, the following individual factors were assessed at the time point just before the beginning of IMiD treatment: family history of thrombosis, age, smoking status, sex, Eastern Cooperative Oncology Group (ECOG) performance status, and relevant comorbidities, such as hypertension, digestive diseases, hepatitis B, stroke, hyperlipidaemia, rheumatic diseases, abnormal pulmonary function, tuberculosis, and other malignancies. The disease-related factors before starting IMiD treatment were assessed and included the International Staging System (ISS) stage, the type of M-protein, the disease stage, the treatment status, and whether the myeloma was combined with amyloidosis. The MM patients were classified as having newly diagnosed MM (NDMM) or refractory or relapsed MM (RRMM) during the course of IMiDs. The treatment status during IMiD use was classified as induction or maintenance therapy. The treatment regimens, such as IMiDs singly or in conjunction with other drugs, erythropoietin, estrogen, and radiation, were determined based on a − 30- to + 30-day window based on the time of IMiD prescription initiation and concurrent anticoagulant and antiplatelet medications were determined utilizing a − 30- to + 7-day window from the start of IMiD prescriptions. Concurrent autologous stem cell transplantation (ASCT) was defined using a retrospective window of up to 12 months from when the IMiD prescription was initiated. For patients who had numerous laboratory results, we chose the values that were acquired nearest to the time of the commencement of IMiDs, including the haemoglobin (Hb) and cholesterol (CHOL) concentrations, the leukocyte and platelet counts, the levels of fibrinogen (FDP) or D-dimer, creatinine (Cr), albumin (ALB), lactate dehydrogenase (LDH), the activated partial thrombin time (APTT), β2-microglobulin (BM2G), triglycerides (TG), low-density lipoprotein (LDL), and the prothrombin time (PT).

### Statistical analysis

R software (version: 3_6.1) and SPSS Statistics (version: 22) was employed to conduct all the statistical analyses. Continuous data were expressed as the mean ± standard deviation (SD) or median [interquartile range, (IQR)] and were subjected to comparisons with the aid of the Mann–Whitney U test or Student’s t-test; categorical data were summarized as percentages (%) and were subjected to comparisons with the aid of the chi-square test or Fisher’s exact test. In addition, we performed a Kaplan–Meier survival analysis for time-dependent factors from the commencement of IMiD therapy until the onset of VTE to determine the trends of VTE progression. For all two-sided statistical tests, significance was determined by a P value of < 0.05.

We used univariate Cox regression models to probe the correlation between an extended collection of risk variables and VTE progression to build a new RAM. A backward stepwise technique with a 0.05 threshold P value was utilized to select the significant variables for the multivariate Cox regression model. As part of the multivariate Cox regression model, the most significant factors were included in the RAM, where integer values were allocated with the aid of the hazard ratio (HR). For both external and internal validation, Harrell’s C indices were employed in conjunction with 200 bootstrap samples to establish the 95% confidence interval (95%CI) for the model’s discrimination. The Kaplan–Meier curve was utilized to estimate the cumulative incidence of VTE during the initial 6 and 12 months after the initiation of IMiD therapy, classified by the IMWG guidelines model, SAVED risk model, and the new RAM. The HR and the P value were used to assess the performance of the risk group. To conduct sensitivity analyses, we excluded patients receiving prophylactic anticoagulation therapy and then calculated the score discrimination using Harrell’s C statistic in both the derivation and validation cohorts. The competing risk was not considered due to the low incidence of early deaths (4.7%) before VTE during the study follow-up.

## Results

### Clinical features of the included patients

Table 1 illustrates the patients’ baseline clinical features. We enrolled 1497 MM patients from 16 major academic medical centers in China from January 2010 to March 2020. All patients received treatment with immunomodulatory drugs (IMiDs). As a result, 163 patients were excluded due to incomplete information, using IMiDs for less than 7 days (because of unsatisfactory effects or other intolerable side effects), receiving therapeutic anticoagulation at the beginning of IMiD treatment or having a previous diagnosis of VTE within 30 days of the first IMiD prescription. Eventually, 1334 patients were included in the analysis for the present study (Fig. 1). The baseline clinical characteristics of the patients are listed (for both the cohorts and the total patients) in Table 1. No data were missing in the baseline variable assessment. In terms of the demographic characteristics and the majority of clinical and laboratory data, there was a similarity between the patients in both the derivation and validation cohorts.

**Table 1:** Baseline characteristics of the derivation and external validation cohorts

	Total patients (N = 1334), n (%)	Derivation cohort (N = 667), n (%)		Validation cohort (N = 667), n (%)
Male sex	753 (56.5)	362 (54.3)		391 (58.6)
Age	61.7 (22.0–96.0)	61.38 (22.0–92.0)		62.03 (28.0–96.0)
BMI	23.1 (13.4–32.1)	23.2 (15.2–31.8)		23.1 (13.4–32.1)
Smoking status	268 (20.1)	137 (20.5)		131 (19.6)
ECOG ≤ 2	671 (50.3)	331 (49.6)		340 (50.9)
History of VTE	46 (3.5)	24 (3.6)		22 (3.3)
Family history of thrombosis	24 (1.8)	6 (0.9)		18 (2.7)
Relevant comorbidities				
Hypertension	338 (25.3)	174 (26.1)		164 (24.6)
Diabetes	123 (9.2)	65 (9.7)		58 (8.7)
Coronary heart disease	47 (3.5)	26 (3.9)		21 (3.1)
Heart disease	50 (3.8)	18 (2.7)		32 (4.8)
Stroke	29 (2.2)	14 (2.1)		15 (2.2)
Recent fracture	123 (9.2)	68 (10.2)		55 (8.2)
Hyperlipidaemia	28 (2.1)	17 (2.5)		11 (1.6)
Rheumatic diseases	10 (0.8)	4 (0.6)		6 (0.9)
COPD or abnormal pulmonary function	63 (4.7)	23 (3.4)		40 (6)
Recent infection	38 (2.9)	16 (2.4)		22 (3.3)
Digestive diseases	13 (1.0)	7 (1)		6 (0.9)
Hepatitis B	58 (4.4)	27 (4)		31 (4.6)
Tuberculosis	21 (1.6)	13 (1.9)		8 (1.2)
Other malignancy	32 (2.4)	19 (2.8)		13 (1.9)
Estrogen	2 (0.1)	2 (0.3)		0 (0)
Erythropoietin	48 (3.6)	21 (3.1)		27 (4.0)
Radiation	10 (0.8)	6 (0.9)		4 (0.6)
Recent immobilization	80 (6.0)	37 (5.5)		43 (6.4)
Central venous catheter or pacemaker	276 (20.7)	136 (20.4)		140 (20.9)
ISS stage				
I	220 (16.5)	117 (17.5)		103 (15.4)
II	509 (38.2)	253 (37.9)		256 (38.4)
III	605 (45.4)	297 (44.5)		308 (46.2)
M protein type				
Light chain	304 (22.8)	148 (22.2)		156 (23.4)
Nonlight chain	1030 (77.2)	519 (77.8)		511 (76.6)
IgG	719 (53.9)	357 (53.5)		362 (54.3)
Non-IgG	615 (46.1)	310 (46.5)		305 (45.7)
Combined with amyloidosis	44 (3.3)	17 (2.5)		27 (4.0)
Disease stage				
NDMM	1096 (82.2)	554 (83.1)	542 (81.3)	
RRMM	238 (17.8)	113 (16.9)	125 (18.7)	
Treatment stage				
Induction therapy	793 (59.5)	388 (58.2)	405 (60.7)	
Maintenance therapy	541 (40.6)	279 (41.8)	262 (39.3)	
ASCT eligible	191 (14.3)	94 (14.1)	97 (14.5)	
IMiD type				
Thalidomide	871 (65.3)	436 (65.4)	435 (65.2)	
Lenalidomide	463 (34.7)	231 (34.6)	232 (34.8)	
Concurrent anticoagulant or antiplatelet drug				
Antiplatelet drug	507 (38.0)	251 (37.6)	256 (38.4)	
Anticoagulant	45 (3.4)	22 (3.3)	23 (3.4)	
Concurrent chemotherapy				
Single	332 (24.9)	175 (26.2)	157 (23.5)	
Dexamethasone	1002 (75.1)	492 (73.8)	510 (76.5)	
Bortezomib	403 (30.2)	207 (31.0)	196 (29.4)	
Cyclophosphamide	245 (18.4)	126 (18.9)	119 (17.8)	
Doxorubicin	209 (15.7)	111 (16.6)	98 (14.7)	
Multiagent (≥ 3 drugs)	673 (50.5)	341 (51.1)	332 (49.8)	
Laboratory data				
PT (s)	12.63 (7.00-26.50)	12.68 (7.00-26.50)	12.58 (7.00-25.50)	
APTT (s)	29.53 (11.10–77.00)	29.61 (14.5–77.00)	29.44 (11.10–73.20)	
FIB (g/L)	3.17 (0.10–9.50)	3.14 (0.10–9.50)	3.19 (0.29–8.38)	
Dimer (mg/L FEU)	1.87 (0.03-64.00)	1.89 (0.03-39.0)	1.85 (0.07-64.00)	
WBC (*10^9^/L)	5.25 (1.10-34.98)	5.24 (1.10–23.20)	5.27 (1.27–34.98)	
Hb (g/L)	99.41 (32.20–193.00)	99.52 (32.20–165.00)	99.29 (33.00-193.00)	
PLT (*10^9^/L)	181.93 (11.00-1329.00)	180.90 (11.00-1329.00)	182.99 (20.00-1329.00)	
Cr (µmol/L)	111.69 (16.20–1162.00)	108.66 (25.00-1162.00)	114.68 (16.20–951.00)	
ALB (g/L)	35.75 (12.00-49.90)	35.74 (12.00-49.90)	35.77 (15.90–48.70)	
LDH (U/L)	194.67 (48.00-974.00)	200.82 (48.00-974.00)	188.50 (76.00-615.00)	
BM2G (µg/L)	5499.84 (260.00-58000.00)	5463.79 (260.00-58000.00)	5535.70 (320.00-43777.62)	
LDL (mmol/L)	2.54 (0.32–11.33)	2.53 (0.32–11.28)	2.54 (0.35–11.33)	
TG (mmol/L)	2.24 (0.16–20.83)	2.27 (0.16–16.72)	2.22 (0.27–20.83)	
CHOL (mmol/L)	4.40 (0.91–24.35)	4.42 (0.96–24.35)	4.38 (0.91–17.44)	
VTE	82 (6.1)	41 (6.1)	41 (6.1)	
Lower extremity	65 (4.9)	34 (5.1)	31 (4.6)	
Pulmonary embolism	6 (0.4)	3 (0.4)	3 (0.4)	
Upper extremity	11 (0.8)	4 (0.6)	7 (1.1)	

Among the entire cohort, 56.45% were male, and their ages ranged from 22 to 96 years, with a median age of 61.7 years. The median BMI was 23.1 kg/m2. At the time of diagnosis, 220 (16.49%), 509 (38.16%), and 605 (45.35%) patients were found to be at stages I, II, and III, respectively, based on the R-ISS. Among the patients having a monoclonality that could be analyzed through the immunofixation of the urine or serum proteins, 719 (53.9%) patients were found to have IgG monoclonal chains; 304 (22.79%) patients were found to only have monoclonal light chains. Regarding the initial treatments, the disease status at the initiation of IMiD treatment was classified into two categories: 1096 (82.16%) patients received IMiDs for NDMM, while 238 (17.84%) received IMiDs for RRMM. Forty-six patients had a previous history of VTE, and 24 patients reported having a family history of thrombosis. A total of 663 (49.70%) patients were shown to have an ECOG performance level of 3 or 4 and were classified as nonmobile. No patients in the present research were prescribed high doses of dexamethasone (> 480 mg monthly) or had a blood clotting condition diagnosed on their medical records.

The median number of months patients were exposed to IMiD was 7.54 months (range 0.23–180.01 months). Thalidomide was the most frequent IMiD given to patients within this cohort (the percentage of patients who received thalidomide or lenalidomide was 65.29% and 43.71%, respectively). 332 patients received IMiD therapy with a single agent, and 1002 (75.11%) received concomitant steroids. Doxorubicin, bortezomib, cyclophosphamide, and ASCT treatments were administered to 209 (15.67%), 403 (30.21%), 245 (18.37%), and 191 (14.32%) patients, respectively. Erythropoietin was used in 48 (3.6%) patients during IMiD treatment; radiotherapy was used in 10 (0.75%) patients.

Eighty-two (6.1%) of the patients developed new VTE after IMiD initiation, and 41 patients developed VTE from the derivation cohort, the same as in the validation cohort. Among the 82 observed cases of VTE, 65 were in the lower extremities, 6 occurred in the pulmonary system, and 11 occurred in the upper extremities. Among the 82 patients who developed VTE, the time to VTE development ranged from 0.23 to 40.0 months, with a median time of 5.98 months. At the end of the 12-month follow-­up period for VTE assessment, 63 patients had died (4.7%) before VTE, and 94 patients were lost to follow-up. The cumulative incidence of VTE in the entire cohort was 5.5% (4.1-6.8%) at 6 months and 7.1% (5.4-8.7%) at 12 months. No patients died from thrombotic or bleeding events. Overall, VTE prophylaxis was given to 552 (41.4%) patients. Specifically, 507 patients received antiplatelet drugs, and 45 received prophylactic anticoagulants. Following the IMWG guidelines, the patients underwent assessment, identifying that 40.0% had a high risk of VTE, while 60.0% were classified as low-risk. Only 4.9% of high-risk patients received anticoagulant agents, and only 37.1% of low-risk patients were given antiplatelet drugs. Based on the SAVED score, only 1.0% of patients were classified as having a high risk of VTE. Anticoagulant agents were not administered to any of the high-risk patients.

### Independent risk variables for VTE in the derivation cohort

Supplementary Table 2 depicts the associations between VTE progression and the laboratory, clinical and demographic parameters in the two cohorts. The significant risk variables for VTE in the derivation obtained based on the univariate analysis included diabetes, ECOG performance status, the use of an erythropoietin-stimulating agent, the use of dexamethasone, and a family history of thrombosis or a history of VTE. No laboratory variables were discovered to have a remarkable correlation with the progression of VTE. Furthermore, the use of anticoagulants or antiplatelet drugs did not affect the development of VTE. A backward stepwise multivariate regression Cox model for the multivariate model was used to integrate all of these factors into one final model. Finally, the multivariate analysis revealed the following significant variables: the use of dexamethasone (HR: 5.51, P = 0.002); diabetes (HR: 2.41, P = 0.016); ECOG performance status > 2 (HR: 2.29, P = 0.015); the use of an erythropoietin-stimulating agent (HR: 6.24, P < 0.001); and a VTE history or a family history of thrombosis (HR: 5.00, P < 0.001).

### Derivation and validation of the new RAM

Using the multivariate analysis, a novel RAM score that is easier to use was established. Point values were allocated to the significant factors depending on the HR as determined by the multivariate Cox regression analysis: dexamethasone scored 3 points, diabetes scored 1 point, an ECOG performance status of > 2 scored 1 point, erythropoietin-stimulating agent scored 3 points, a VTE history or a family history of thrombosis scored 3 points (Table 2). Depending on the weighting of each variable based on its respective HR, we generated an ordinal score ranging from 0 to 11.

**Table 2:** Derivation and validation of the new RAM score using multivariate Cox regression analysis

Variable	Derivation cohort		Validation cohort		Point value
	HR	P value	HR	P value	
Dexamethasone	5.511	0.002	17.304	0.005	3
Diabetes	2.412	0.016	3.037	0.006	1
Erythropoietin	6.241	< 0.001	3.122	0.018	3
History of VTE or family history of thrombosis	5.001	< 0.001	1.819	0.233	3
ECOG > 2	2.288	0.015	2.397	0.011	1
Risk stratification					
High/low	6.08	< 0.001	6.23	< 0.001	High if > 4
C index	0.64	95% CI: 0.60–0.69	0.62	95% CI: 0.59–0.66	

**Fig. 2:**
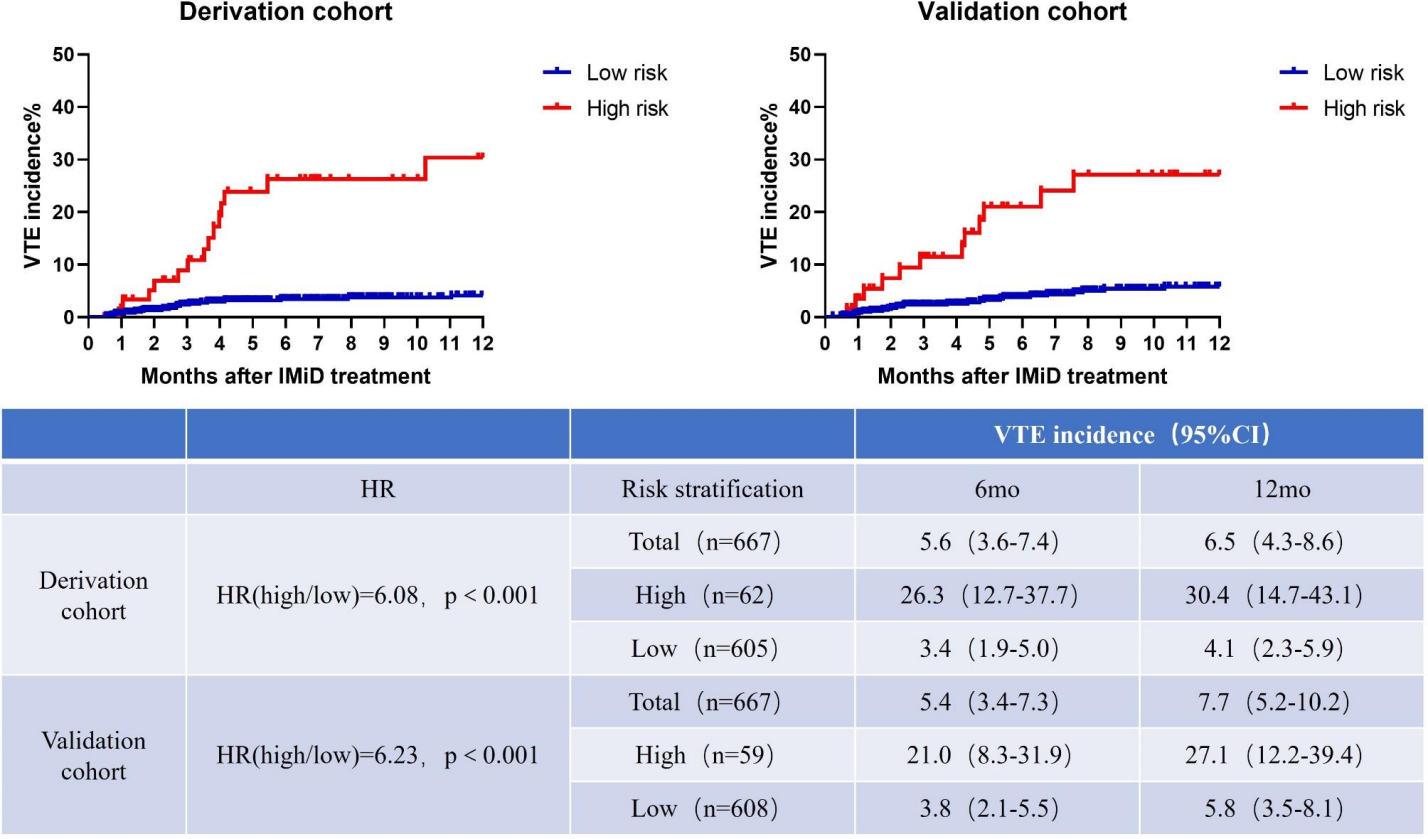
VTE incidence and Kaplan‒Meier curves of patients stratified by the new RAM. Abbreviations: HR, hazard ratio; IMiD, immunomodulatory drug; RAM, risk assessment model; VTE, venous thromboembolism

The novel RAM score was used to stratify the patients into two risk cohorts to identify those most at risk for VTE. Sixty-two patients (9.2%) in the derivation cohort whose scores were > 4 were classified into the high-risk cohort, whereas 605 patients (90.8%) whose scores were ≤ 4 were classified into the low-risk cohort. Among those at high risk for VTE, the incidences were 26.3% (12.7-37.7%) and 30.4% (14.7-43.1%) at 6 and 12 months, respectively; however, among those at low risk for VTE, the incidences were 3.4% (1.9-5.0%) and 4.1% (2.3-5.9%) at 6 and 12 months, respectively (Fig. 2). Among those who had VTE, the hazard ratio (HR) was 6.08 (P < 0.001), and the C index was 0.64 (range: 0.60–0.69) (Table 2).

After external validation of the RAM with the validation cohort, the same risk factors were found to have comparable distributions and magnitudes in both cohorts. The new RAM model stratified 59 patients (8.8%) as high risk for VTE and 608 patients (91.2%) as low risk for VTE. The matching HR for VTE was 6.23 (P < 0.001) in the high-risk cohort in contrast with the low-risk cohort, and the C index was 0.62 (0.59–0.66). The current model performed well in calibration and had similarly expected and observed VTE events at different time points (Fig. 2).

Utilizing derivation and validation datasets, we conducted a sensitivity analysis. First, we investigated the prediction accuracy in the derivation dataset after eliminating the patients receiving anticoagulant medication for any reason at baseline. In a subgroup of 645 patients who did not receive anticoagulant therapy, the C index was found to be 0.64, and the HR was 6.13 (P = 0.001) for the high-risk cohort compared to the low-risk cohort. After eliminating patients taking anticoagulant treatment from the validation cohort, a C statistic of 0.63 and an HR of 6.17 (P < 0.001) was obtained.

### Assessment of the performance of the IWMG guidelines

**Fig. 3:**
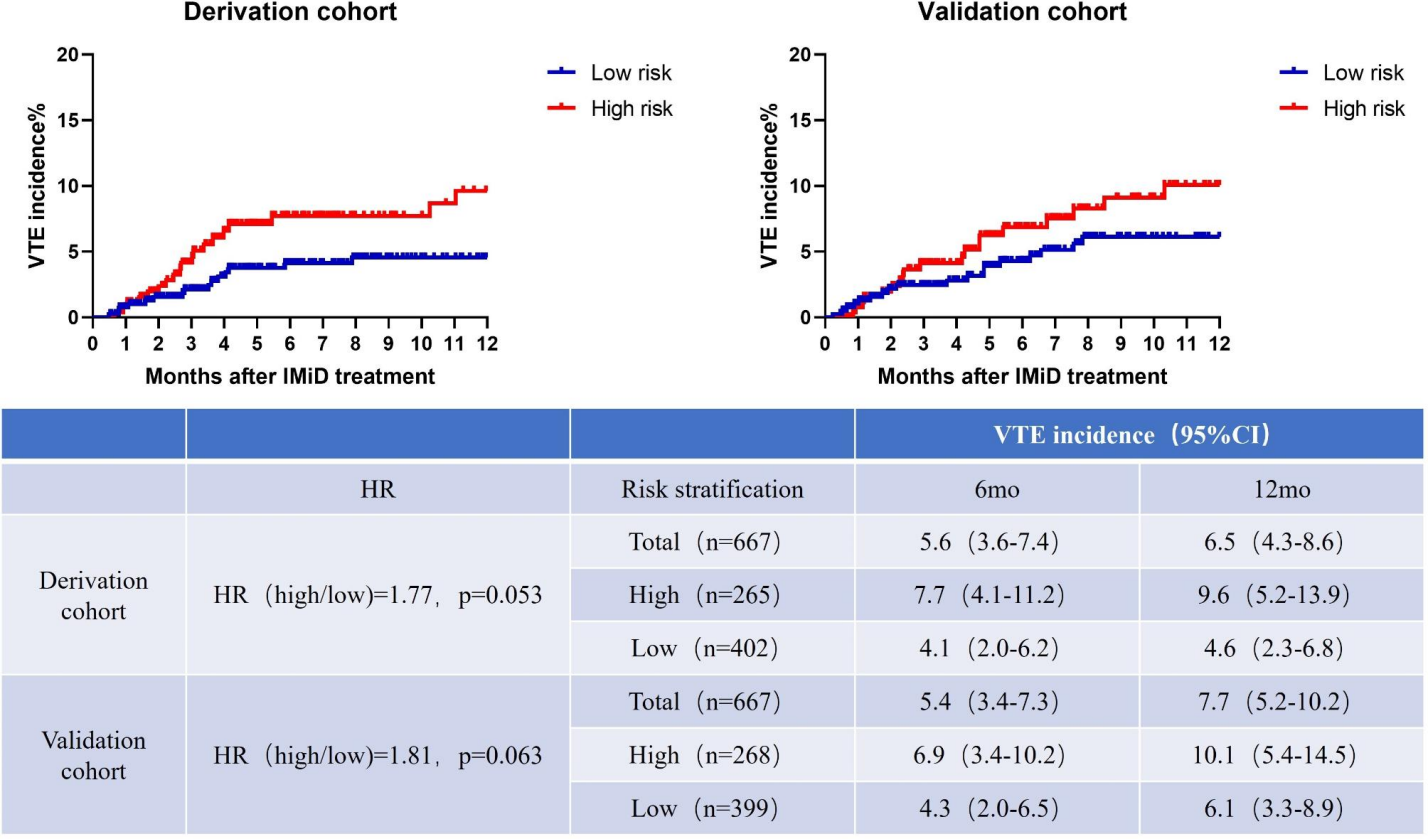
VTE incidence and Kaplan‒Meier curves of patients stratified by the IMWG guidelines. Abbreviations: HR, hazard ratio; IMiD, immunomodulatory drug; RAM, risk assessment model; VTE, venous thromboembolism

In both cohorts, we evaluated the performance of the IMWG guidelines (Table 3). The guidelines classified 265 patients (39.7%) with MM in the derivation dataset into a high-risk group and 402 patients (60.3%) into a low-risk group. According to the IMWG guidelines, the incidence rates of VTE at 6 and 12 months were 7.7% (4.1-11.2%) and 9.6% (5.2-13.9%), respectively, in the high-risk group, whereas they were 4.1% (2.0-6.2%) and 4.6% (2.3-6.8%), respectively, in the low-risk group, indicating that the guidelines failed to anticipate the early occurrence of VTE in a correct manner (HR, 1.77; P = 0.053; C index = 0.58) (Fig. 3). The IMWG rules had a Harrell’s C statistic of 0.55 in this validation sample. The VTE risk in high-risk patients (≥ 2 points) vs. low-risk patients (0–1 points) was 1.81 (P value = 0.063).

The sensitivity analysis model (wherein patients who had previously been treated using prophylactic anticoagulants (n-22) were omitted) was found to have an HR of 1.68 (P = 0.109) for the high-risk group in contrast with the low-risk group and a C index of 0.58. The analysis was repeated using the validation cohort (HR = 1.91, p = 0.048, C index = 0.57) (Fig. 3; Table 3).

### Assessment of the performance of the SAVED score

Table 3 presents the five SAVED variables and HRs. Our study revealed a significant association between VTE history and dexamethasone dose. Based on the SAVED score, 6 patients (0.9%) were classified as high-risk and 661 (99.1%) low-risk. In the group at high risk, the cumulative incidence of VTE at six and 12 months was 25.0% (0-57.4%) and 25.0% (0-57.4%), respectively, whereas in the low-risk group, it was 5.4% (3.5-7.2%) and 6.3% (4.1-8.4%), respectively. Moreover, the SAVED score demonstrated inadequate predictive ability for the early occurrence of VTE in a correct manner (HR, 3.23; P = 0.248; C index = 0.51). In this validation cohort, the SAVED score had a Harrell’s C statistic of 0.50. The incidence of VTE was 1.2% in high-risk patients (n = 8) and 98.8% in low-risk patients (n = 659), with a calculated risk ratio of 1.65 (P = 0.622). (see Supplementary Fig. 1)

The sensitivity analysis model excluded patients who had previously received prophylactic anticoagulants (n = 22). The model showed an HR of 3.19 (P = 0.252) for the high-risk group compared to the low-risk group and a C index of 0.51. The analysis was repeated using the validation cohort (HR = 1.71, p = 0.598, C index = 0.50).

**Table 3:** Univariate Cox regression analysis of IMWG guideline performance and SAVED scores

IMWG guideline performance				
**Proposed risk factor**	**Derivation cohort**		**Validation cohort**	
	**HR**	**P value**	**HR**	**P value**
Individual				
Prior VTE	2.970	0.039	0.618	0.634
Obesity	0.897	0.882	1.165	0.833
Central venous catheter or pacemaker	0.860	0.717	1.255	0.532
Cardiac disease (CHF, MI, CA)	0.048	0.47	1.552	0.464
Chronic renal disease	1.717	0.224	0.693	0.541
Diabetes	2.919	0.003	2.812	0.009
Acute infection	0.048	0.483	0.047	0.413
Immobilization	0.726	0.659	0.614	0.502
Trauma or surgery	0.981	0.985	0.781	0.807
Blood clotting disorders	NE	NE	NE	NE
Erythropoietin	5.310	0.001	3.725	0.006
Myeloma-related				
Diagnosis of myeloma per se	N/A	N/A	N/A	N/A
Hyperviscosity	N/A	N/A	N/A	N/A
Therapy-related				
High-dose dexamethasone, ≥ 480 mg	NE	NE	NE	NE
Doxorubicin	2.197	0.026	2.137	0.037
Multiagent (cytotoxic) chemotherapy	0.688	0.304	0.81	0.577
Risk stratification				
High/low	1.77	0.053	1.81	0.063
C index	0.58	95% CI: 0.54–0.63	0.55	95% CI: 0.51–0.60
**SAVED scores**				
**Proposed risk factor**	**Derivation cohort**		**Validation cohort**	
	**HR**	**P value**	**HR**	**P value**
Surgery (within 90 days)	0.640	0.478	0.819	0.742
Asian race	NE	NE	NE	NE
VTE history	4.378	0.008	1.108	0.920
Eighty (age ≥ 80y)	1.072	0.924	0.962	0.957
Dexamethasone dose				
Standard dose (120-160 mg)	5.404	0.002	16.737	0.006
High dose (>160 mg)	NE	NE	NE	NE
Risk stratification				
High/low	3.225	0.248	1.647	0.622
C index	0.51	95% CI: 0.49–0.52	0.50	95% CI: 0.49–0.51

## Discussion

VTE is a serious medical disorder and complication that can occur in individuals with MM undergoing IMiD treatment. As IMiD treatment remains a cornerstone of MM therapy, precise risk stratification for VTE is paramount. The risk stratification of multiple myeloma (MM) patients treated with immunomodulatory drugs (IMiDs) is mainly determined by the IMWG guideline, SAVED score, or IMPEDE-VTE score. The SAVED model was specifically developed for MM patients receiving IMiD. Most of the guidelines were developed in Western countries. However, individuals of Asian race have been found to have a lower risk of VTE. Although ethnic factors were considered, the guidelines did not fully consider ethnic differences. The sample size of Asian participants in the sample (SAVED), which was merely 7%, was relatively small. Furthermore, no independent external validation of this risk model has been conducted in China [13, 14, 22, 23]. In this study, we analyzed a large multicentre dataset of MM patients who received IMiD treatment in China, and this study represents MM patients treated across the country. The present research findings revealed that the incidence of VTE in Chinese MM patients had at least three different characteristics. One important finding of the present research was a low VTE incidence (6.1%) in Chinese MM patients treated with IMiDs, despite the absence of thromboprophylaxis, which is consistent with previous reports within Asian populations [20, 24]. However, these results are lower than those of previous studies from Western countries [25], where the VTE incidences were highly variable among the different trials, and the incidence rate of VTE could be as high as 58% when IMiDs were given without thromboprophylaxis [26, 27].

In addition, one of the most significant findings of this study was that the most of risk variables and factors associated with VTE incidence in Chinese subjects differed from those previously reported in Western studies and outlined in the IMWG guidelines and SAVED score. Therefore, it is reasonable to hypothesize that the pathogenesis of VTE in Chinese patients differs from that in Western patients, which aligns with the lower frequencies and distinct patterns of VTE progression observed in our study compared to Western studies. It is possible that other factors, including nutrition and prothrombin mutations, lead to a lower incidence rate of VTE among the overall Asian population [28, 29].

The third finding of our study is that VTE prophylaxis (including antiplatelet and anticoagulant drugs) was provided to only 41.38% of the patients in the study cohort, which is lower in contrast with the percentages in Western nations. Most of the patients received antiplatelet drug prophylaxis (38.01%), and other prophylactic therapies, including low-molecular-weight warfarin or heparin, were utilized in a limited number of Chinese patients. According to the IMWG guidelines, 40.0% of the patients in our study were classified as having a high risk for VTE, whereas 60.0% were classified as having a low risk for VTE. Anticoagulant agents were given in only 26 (4.9%) high-risk patients, and antiplatelet drugs were given in 297 (37.1%) low-risk patients. According to the SAVED score, only 1.0% were classified as having a high risk for VTE, and none of these high-risk patients were administered anticoagulant agents. The above data indicate that prevention strategies for VTE in Chinese patients are based on Chinese expert experiences rather than IMWG guidelines or SAVED scores. However, the use of antiplatelet drugs or anticoagulation therapies may not reduce the risk of VTE in the Chinese population based on Chinese expert experiences in this study (as shown in supplementary Tables 2, VTE with or without antiplatelet drugs: 7.2% and 5.5% (p = 0.392), and anticoagulation therapies: 9.1% and 6.0% (p = 0.636), respectively), which is consistent with previous reports from Korea and Japan, but inconsistent with some results reported in Western myeloma patients treated with IMiDs [18, 20, 30]. The ineffectiveness of anticoagulant and antiplatelet therapy in preventing VTE may be due to the failure to use prophylactic drugs in patients with genuine high risk.

The above three differences showed that the rate of VTE is lower in Chinese myeloma patients compared with Western patients. The current guidelines, such as IMWG and SAVED, include several factors that are not relevant to the incidence of VTE among Chinese subjects in the present study. Moreover, VTE prevention based on the experience of Chinese experts rather than on precise risk stratification guidance may not effectively prevent VTE. Therefore, more precise risk stratification is required for VTE in China. To address this particular clinical need, we analyzed a large and nationally representative multicentre dataset that included MM patients in China who had IMiD treatment, and we used this new model to develop a new RAM that was capable of predicting the risk of IMiD-related VTE in MM patients. This model comprised the following five variables: diabetes; ECOG performance status; the use of an erythropoietin-stimulating agent; the use of dexamethasone; and a VTE history or a family history of thrombosis. Furthermore, we externally and independently validated the new RAM and confirmed its generalizability and robust predictive performance. This is the first clinical RAM validated in China for MM patients receiving IMiDs. It has been shown that the discriminative performance of this novel RAM model, which contains just five factors, is better than that of the more sophisticated consensus model proposed by the IMWG guidelines.

In this cohort, we found that diabetes, erythropoietin-stimulating agent use, dexamethasone use, and VTE history were independent predictors of IMiD-related VTE, which is in agreement with most previous studies, and our results are also in agreement with the IMWG guidelines for the risk variables correlated with IMiD-related VTE. Furthermore, dexamethasone use and a history of VTE as predictors are consistent with the SAVED scores. In previous research, it was found that when thalidomide and dexamethasone or lenalidomide and dexamethasone were used in a combined manner, the incidence increased to 17% and that the incidence increased even more to 26–58% in patients who were also given further chemotherapy treatment [25, 31]. Furthermore, it has been observed that administering an erythropoiesis-stimulating drug in conjunction with lenalidomide and dexamethasone can elevate the risk of VTE from 5 to 23% in individuals receiving the combination therapy [32]. Furthermore, our study revealed that the ECOG performance status and family history of thrombosis, variables not incorporated into the IMWG guidelines and SAVED scores, had significant predictive value as independent factors. Some previous studies have shown that a family history of thrombosis independently serves as a risk variable for VTE in 12 cancers, including MM [33]. Patients with decreased ECOG performance scores may represent a more chronically ill population, and a decreased ECOG performance status may be more prevalent in MM patients with a high risk of VTE. This finding was similar to our previous MM studies [34, 35].

The present research found no significant predictors of VTE for a number of characteristics that had previously been correlated with an elevated risk of VTE in other groups. Patient-related risk and treatment-related factors, including age and male sex, concomitant infections, immobility, obesity, major illnesses (chronic renal disease, inflammatory bowel disease, autoimmune diseases, chronic obstructive pulmonary disease, cardiovascular disease, congestive cardiac failure), surgery, radiation, fractures, central venous catheters, and hormonal therapy, are critical cofactors in the pathophysiology of thromboses and were not correlated with the VTE risk in the sample of the present research, which is in contrast to prior studies [36–38].

In our cohort of Chinese patients, we compared the performance of IMWG guidelines and SAVED score assessment models for VTE in MM patients treated with IMiDs. Our results revealed that IMWG guidelines and SAVED scores failed to predict patients at risk for VTE development among our Chinese sample. Although approximately 39.7% of the 667 patients with MM in our derivation cohort could have been designated as “high thrombotic risk” based on the available IMWG consensus, the IMWG model failed to predict the initial VTE onset accurately; with this model, the incidences of VTE at 6 and 12 months were 7.7% (4.1-11.2%) and 9.6% (5.2-13.9%), respectively, in the high-risk group and 4.1% (2.0-6.2%) and 4.6% (2.3-6.8%), respectively, in the low-risk group (HR, 1.77; P = 0.053; C index = 0.58) (Fig. 3; Table 3). Based on the IMWG guidelines, 39.7% of the patients who had been classified as “high thrombotic risk” should be treated with anticoagulation therapy to prevent VTE, but these therapies may not effectively prevent thrombosis and may increase the financial burden and the risk of bleeding.

The SAVED score [15] has recently been incorporated into the NCCN guidelines. It was introduced as a simpler method for VTE prediction among MM patients and took into consideration only five variables. However, the SAVED score could not effectively classify patients in our cohort into high and low-risk categories. Out of all patients, 6.1% have VTE. However, according to the SAVED score, only 1% of patients are identified as high-risk, indicating an overall low discrimination performance with a Harrell’s c-statistic of 0.51, which is even inferior to the predictive power of IMWG guidelines. The number of patients classified as high-risk following the SAVED score was too small in our study. The reasons for this result include the following: All the patients in our cohort are of Asian ethnicity. The median age of patients in the SAVED score development study was 74 years, which was higher than that of our cohort. Although a high dose of dexamethasone is considered a predictor in the SAVED score, none of the patients in our Chinese cohort exhibited this characteristic. Therefore, using the SAVED score for VTE risk stratification may underestimate the risk of VTE in Chinese patients.

In our new RAM score, we used only five variables to differentiate between the high- and low-risk patients. A total of 62 patients (9.2%) with a score > 4 were defined as high-risk patients, and 605 patients (90.8%) with a score of ≤ 4 were categorized as low-risk patients. In the high-risk category, the incidence rates of VTE were 26.3% (12.7-37.7%) and 30.4% (14.7-43.1%) at 6 and 12 months, respectively, and in the low-risk category, the rates were 3.4% (1.9-5.0%) and 4.1% (2.3-5.9%) at 6 and 12 months, respectively (Fig. 2). The HR for VTE was 6.08 (P < 0.001), with a C index of 0.64 (0.60–0.69) (Table 2). In the present research, the superior clinical scores comprised C indices ranging from 0.58 to 0.64 within the validation cohort, and these parameters made the prediction model more accurate, sensitive, and effective. After excluding the patients who had anticoagulation therapy, a sensitivity analysis of the derivation and validation cohorts showed that the model was still capable of discriminating the risk with C indices of 0.64 and 0.63.

To the best of our knowledge, prediction of IMiD-related VTE has never been reported in Chinese MM patients, and the recently developed RAM score is the first tool for this purpose. Our new model has demonstrated superior performance compared to the IMWG guidelines and SAVED score. We successfully identified a “highest-risk” group of patients who ought to be treated with primary anticoagulant thromboprophylaxis, which is more aggressive than aspirin therapy at the onset of IMiD treatment. This simplified set of clinical risk predictive variables could function as novel guidelines for assessing innovative prognostic biological indicators among this high-risk population of patients. Medical physicians can gain insight from our innovative RAM rating system to establish the most effective treatment plan for each patient and to enhance their patient counseling efficiency. Nevertheless, several limitations exist in the present research. The retrospective research methodology made it difficult to determine whether biological gene markers might enhance the discrimination of scores. Even though the present research included the largest dataset of MM patients undergoing IMiD therapy in China, our proposed model still needs additional exploration and validation. Secondly, after this study was concluded, the FDA approved additional chemotherapeutic agents for the treatment of MM (e.g., pomalidomide, carfilzomib, ixazomib, daratumumab); therefore, the association of these agents with VTE was not analyzed in our study. Thirdly, as this is a retrospective cohort study, we do not routinely perform imaging tests or structured assessments to detect thrombosis in our centers for MM patients who have used IMiD treatment unless they exhibit thrombotic-related symptoms. Asymptomatic VTE may have been neglected, which increases the possibility of missing VTE episodes in our cohort.

## Conclusions

In conclusion, the use of IMiD therapy in Chinese patients with MM was associated with a low incidence of VTE. Current treatments for thrombosis lack effectiveness due to the difficulty in accurately predicting patients at an elevated VTE risk. We developed and validated a distinctive RAM score that demonstrated superior performance compared to the risk stratification established by the IMWG guidelines and SAVED score. When a patient has a high risk of VTE, risk assessment might assist practitioners in prescribing thromboprophylaxis and preventing the use of anticoagulant in patients who have a reduced VTE risk. These findings suggest that the innovative RAM score could replace the current risk stratification standards for identifying MM patients with high VTE risk in China.

### Electronic supplementary material

Below is the link to the electronic supplementary material.

Supplementary Material 1

Supplementary Material 2

## Data Availability

The data sets analysed in the current study are available from the corresponding author on reasonable request.

## Abbreviations

VTE: Venous thromboembolism
MM: Multiple myeloma
IMiD: Immunomodulatory drug
RAM: Risk assessment model
ECOG: Eastern Cooperative Oncology Group
NDMM: Newly diagnosed MM
RRMM: Refractory or relapsed MM
ASCT: Autologous stem cell transplantation
