# Supplementary material for: Development and validation of a new risk assessment model for immunomodulatory drug-associated venous thrombosis among Chinese patients with multiple myeloma
Source: Thromb J. 2023 Oct 4;21:105. doi: 10.1186/s12959-023-00534-y (PMC10552366; doi:10.1186/s12959-023-00534-y)
Supplement: Supplementary file 1 — Supplementary Material 1 [file 12959_2023_534_MOESM1_ESM.docx]

**Supplementary Tables**

**Supplementary Table 1.** The potential risk factors for VTE that were collected, including individual factors, disease information, treatment factors and laboratory parameters.

| IMWG risk factors | Additional risk factors | | | |
| --- | --- | --- | --- | --- |
|  | Individual factor | Disease information | Treatment factor | Laboratory |
| Obesity (> 28 kg/m^2^) | Age, sex | Disease stage | Aspirin | PT (s) |
| Chronic renal disease (GFR < 60) | Smoking status | Treatment status | Anticoagulation | APTT (s) |
| Trauma or surgery | Family history of thrombosis | M-protein at diagnosis | ASCT eligible | FIB (g/L) |
| Prior VTE | ECOG performance status | Amyloidosis | Chemotherapy (single, dexamethasone, bortezomib, cyclophosphamide, doxorubicin, multiagent [≥ 3 drugs]) | Dimer (mg/L FEU) |
| Diabetes | Hypertension |  | IMiD type | WBC (*10^9^/L) |
| Central venous catheter or Pacemaker | Digestive diseases |  | Oestrogen | Hb (g/L) |
| Acute infection | Hepatitis B |  | Radiation | PLT (*10^9^/L) |
| Cardiac disease | Stroke |  |  | Cr (µmol/L) |
| Immobilization | Recent fracture |  |  | ALB (g/L) |
| Erythropoietin | Hyperlipidaemia |  |  | LDH (U/L) |
| High-dose dexamethasone, ≥ 480 mg | Rheumatic diseases |  |  | BM2G (µg/L) |
| Doxorubicin | COPD or abnormal pulmonary function |  |  | LDL (mmol/L) |
| Multiagent (cytotoxic) chemotherapy | Tuberculosis |  |  | TG (mmol/L) |
|  | Other malignancy |  |  | CHOL (mmol/L) |

**Supplementary Table 2.** Univariate Cox regression analysis of the derivation and validation cohorts

|  | Derivation cohort | | | | | Validation cohort | | | | |
| --- | --- | --- | --- | --- | --- | --- | --- | --- | --- | --- |
| Demographic/clinical characteristic | N | No (n=626) | Yes (n=41) | HR | P value | N | No (n=626) | Yes (n=41) | HR | P value |
| Age |  | 61.19±10.61 | 64.20±9.48 |  | 0.078 |  | 62.02±10.53 | 62.15±10.22 |  | 0.939 |
| BMI |  |  |  |  |  |  |  |  |  |  |
| >28 | 30 | 28 (93.3) | 2 (6.7) | 1.110 | 0.882 | 25 | 23 (92.0) | 2 (8.0) | 1.165 | 0.833 |
| ≤28 | 637 | 598 (93.9) | 39 (6.1) |  |  | 642 | 603 (93.9) | 39 (6.1) |  |  |
| Sex |  |  |  |  |  |  |  |  |  |  |
| Male | 362 | 336 (92.8) | 26 (7.2) | 0.676 | 0.227 | 391 | 370 (94.6) | 21 (5.4) | 1.308 | 0.390 |
| Female | 305 | 290 (95.1) | 15 (4.9) |  |  | 276 | 256 (92.8) | 20 (7.2) |  |  |
| Smoking status |  |  |  |  |  |  |  |  |  |  |
| Yes | 137 | 129 (94.2) | 8 (5.8) | 1.028 | 0.944 | 131 | 127 (96.9) | 4 (3.1) | 2.175 | 0.140 |
| No | 530 | 497 (93.8) | 33 (6.2) |  |  | 536 | 499 (93.1) | 37 (6.9) |  |  |
| ECOG |  |  |  |  |  |  |  |  |  |  |
| ≤2 | 331 | 318 (96.1) | 13 (3.9) | 2.290 | 0.014 | 340 | 327 (96.2) | 13 (3.8) | 2.712 | 0.003 |
| >2 | 336 | 308 (91.7) | 28 (8.3) |  |  | 327 | 299 (91.4) | 28 (8.6) |  |  |
| History of VTE or family history of thrombosis |  |  |  |  |  |  |  |  |  |  |
| Yes | 29 | 23 (79.3) | 6 (20.7) | 4.090 | 0.001 | 33 | 28 (84.8) | 5 (15.2) | 2.210 | 0.096 |
| No | 638 | 603 (94.5) | 35 (5.5) |  |  | 634 | 598 (94.3) | 36 (5.7) |  |  |
| Hypertension |  |  |  |  |  |  |  |  |  |  |
| Yes | 174 | 163 (93.7) | 11 (6.3) | 1.046 | 0.898 | 164 | 152 (92.7) | 12 (7.3) | 0.739 | 0.379 |
| No | 493 | 463 (93.9) | 30 (6.1) |  |  | 503 | 474 (94.2) | 29 (5.8) |  |  |
| Diabetes |  |  |  |  |  |  |  |  |  |  |
| Yes | 65 | 55 (84.6) | 10 (15.4) | 2.919 | 0.003 | 58 | 50 (86.2) | 8 (13.8) | 2.812 | 0.009 |
| No | 602 | 571 (94.9) | 31 (5.1) |  |  | 609 | 576 (94.6) | 33 (5.4) |  |  |
| Heart disease |  |  |  |  |  |  |  |  |  |  |
| Yes | 18 | 18 (100.0) | 0 (0) | 0.048 | 0.470 | 32 | 29 (90.6) | 3 (9.4) | 1.552 | 0.464 |
| No | 649 | 608 (93.7) | 41 (6.3) |  |  | 635 | 597 (94.0) | 38 (6.0) |  |  |
| Stroke |  |  |  |  |  |  |  |  |  |  |
| Yes | 14 | 12 (85.7) | 2 (14.3) | 2.440 | 0.219 | 15 | 15 (100.0) | 0 (0.0) | 0.048 | 0.475 |
| No | 653 | 614 (94.0) | 39 (6.0) |  |  | 652 | 611 (93.7) | 41 (6.3) |  |  |
| Recent fracture |  |  |  |  |  |  |  |  |  |  |
| Yes | 68 | 66 (97.1) | 2 (2.9) | 2.307 | 0.249 | 55 | 52 (94.5) | 3 (5.5) | 1.145 | 0.821 |
| No | 599 | 560 (93.5) | 39 (6.5) |  |  | 612 | 574 (93.8) | 38 (6.2) |  |  |
| Hyperlipidaemia |  |  |  |  |  |  |  |  |  |  |
| Yes | 17 | 15 (88.2) | 2 (11.8) | 2.520 | 0.204 | 11 | 10 (90.9) | 1 (9.1) | 1.850 | 0.543 |
| No | 650 | 611 (94.0) | 39 (6.0) |  |  | 656 | 616 (93.9) | 40 (6.1) |  |  |
| Rheumatic diseases |  |  |  |  |  |  |  |  |  |  |
| Yes | 4 | 4 (100.0) | 0 (0) | 0.049 | 0.732 | 6 | 5 (83.3) | 1 (16.7) | 1.115 | 0.163 |
| No | 663 | 633 (93.8) | 41 (6.2) |  |  | 661 | 621 (93.9) | 40 (6.1) |  |  |
| COPD or abnormal pulmonary function |  |  |  |  |  |  |  |  |  |  |
| Yes | 23 | 21 (91.3) | 2 (8.7) | 1.821 | 0.409 | 40 | 36 (90.0) | 4 (10.0) | 1.905 | 0.221 |
| No | 644 | 605 (93.9) | 39 (6.1) |  |  | 627 | 590 (94.1) | 37 (5.9) |  |  |
| Recent infection |  |  |  |  |  |  |  |  |  |  |
| Yes | 16 | 16 (100.0) | 0 (0) | 0.048 | 0.483 | 22 | 22 (100.0) | 0 (0) | 0.047 | 0.413 |
| No | 651 | 610 (93.7) | 41 (6.3) |  |  | 645 | 604 (93.6) | 41 (6.4) |  |  |
| Digestive diseases |  |  |  |  |  |  |  |  |  |  |
| Yes | 7 | 7 (100.0) | 0 (0) | 0.049 | 0.621 | 6 | 6 (100.0) | 0 (0) | 0.049 | 0.664 |
| No | 660 | 619 (93.8) | 41 (6.2) |  |  | 661 | 620 (93.8) | 41 (6.2) |  |  |
| Hepatitis B |  |  |  |  |  |  |  |  |  |  |
| Yes | 27 | 24 (88.9) | 3 (11.1) | 1.866 | 0.298 | 31 | 30 (96.8) | 1 (3.2) | 0.432 | 0.408 |
| No | 640 | 602 (94.1) | 38 (5.9) |  |  | 636 | 596 (93.7) | 40 (6.3) |  |  |
| Tuberculosis |  |  |  |  |  |  |  |  |  |  |
| Yes | 13 | 13 (100.0) | 0 (0) | 0.048 | 0.542 | 8 | 7 (87.5) | 1 (12.5) | 1.818 | 0.556 |
| No | 654 | 613 (93.7) | 41 (6.3) |  |  | 659 | 619 (93.9) | 40 (6.1) |  |  |
| Other malignancy |  |  |  |  |  |  |  |  |  |  |
| Yes | 19 | 17 (89.5) | 2 (10.5) | 1.754 | 0.438 | 13 | 12 (92.3) | 1 (7.7) | 1.055 | 0.958 |
| No | 648 | 609 (94.0) | 39 (6.0) |  |  | 654 | 614 (93.9) | 40 (6.1) |  |  |
| ISS stage |  |  |  |  |  |  |  |  |  |  |
| I | 117 | 113 (96.6) | 4 (3.4) | 1.498 | 0.079 | 103 | 98 (95.1) | 5 (4.9) | 1.858 | 0.014 |
| II | 253 | 237 (93.7) | 16 (6.3) |  |  | 256 | 247 (96.5) | 9 (3.5) |  |  |
| III | 297 | 276 (92.9) | 21 (7.1) |  |  | 308 | 281 (91.2) | 27 (8.8) |  |  |
| Combined with amyloidosis |  |  |  |  |  |  |  |  |  |  |
| Yes | 17 | 16 (94.1) | 1 (5.9) | 0.839 | 0.863 | 27 | 24 (88.9) | 3 (11.1) | 0.409 | 0.137 |
| No | 650 | 610 (93.8) | 40 (6.2) |  |  | 640 | 602 (94.1) | 38 (5.9) |  |  |
| IMiD type |  |  |  |  |  |  |  |  |  |  |
| Thalidomide | 436 | 411 (94.3) | 25 (5.7) | 1.550 | 0.179 | 435 | 412 (94.7) | 23 (5.3) | 1.655 | 0.111 |
| Lenalidomide | 231 | 215 (93.1) | 16 (6.9) |  |  | 232 | 214 (92.2) | 18 (7.8) |  |  |
| Disease stage |  |  |  |  |  |  |  |  |  |  |
| NDMM | 554 | 519 (93.7) | 35 (6.3) | 1.190 | 0.695 | 542 | 510 (94.1) | 32 (5.9) | 0.630 | 0.696 |
| RRMM | 113 | 107 (94.7) | 6 (5.3) |  |  | 125 | 116 (92.8) | 9 (7.2) |  |  |
| Treatment status |  |  |  |  |  |  |  |  |  |  |
| Induction therapy | 388 | 360 (92.8) | 28 (7.2) | 2.300 | 0.016 | 405 | 375 (92.6) | 30 (7.4) | 2.430 | 0.013 |
| Maintenance therapy | 279 | 266 (95.3) | 13 (4.7) |  |  | 262 | 251 (95.8) | 11 (4.2) |  |  |
| ASCT eligible |  |  |  |  |  |  |  |  |  |  |
| Yes | 94 | 92 (97.9) | 2 (2.1) | 4.019 | 0.055 | 97 | 90 (92.8) | 7 (7.2) | 1.092 | 0.833 |
| No | 573 | 534 (93.2) | 39 (6.8) |  |  | 570 | 536 (94.0) | 34 (6.0) |  |  |
| Light chain |  |  |  |  |  |  |  |  |  |  |
| Yes | 148 | 137 (92.6) | 11 (7.4) | 1.393 | 0.347 | 156 | 144 (92.3) | 12 (7.7) | 1.548 | 0.203 |
| No | 519 | 489 (94.2) | 30 (5.8) |  |  | 511 | 482 (94.3) | 29 (5.7) |  |  |
| IgG |  |  |  |  |  |  |  |  |  |  |
| Yes | 357 | 340 (95.2) | 17 (4.8) | 0.547 | 0.058 | 362 | 339 (93.6) | 23 (6.4) | 0.981 | 0.951 |
| No | 310 | 286 (92.3) | 24 (7.7) |  |  | 305 | 287 (94.1) | 18 (5.9) |  |  |
| Estrogen |  |  |  |  |  |  |  |  |  |  |
| Yes | 2 | 2 (100.0) | 0 (0) | 20.166 | 0.854 | 0 | 0 (0) | 0 (0) |  |  |
| No | 665 | 624 (93.8) | 41 (6.2) |  |  | 667 | 626 (93.9) | 41 (6.1) |  |  |
| EPO |  |  |  |  |  |  |  |  |  |  |
| Yes | 21 | 16 (76.2) | 5 (23.8) | 5.310 | 0.001 | 27 | 22 (81.5) | 5 (18.5) | 3.725 | 0.006 |
| No | 646 | 610 (94.4) | 36 (5.6) |  |  | 640 | 604 (94.4) | 36 (5.6) |  |  |
| Radiation |  |  |  |  |  |  |  |  |  |  |
| Yes | 6 | 6 (100.0) | 0 (0) | 20.549 | 0.621 | 4 | 4 (100.0) | 0 (0) | 20.295 | 0.727 |
| No | 661 | 620 (93.8) | 41 (6.2) |  |  | 663 | 622 (93.8) | 41 (6.2) |  |  |
| Immobilization |  |  |  |  |  |  |  |  |  |  |
| Yes | 37 | 34 (91.9) | 3 (8.1) | 1.380 | 0.659 | 43 | 41 (95.3) | 2 (4.7) | 1.630 | 0.502 |
| No | 630 | 592 (94.0) | 38 (6.0) |  |  | 624 | 585 (93.8) | 39 (6.3) |  |  |
| CVC |  |  |  |  |  |  |  |  |  |  |
| Yes | 136 | 128 (94.1) | 8 (5.9) | 0.860 | 0.717 | 140 | 130 (92.9) | 10 (7.1) | 1.255 | 0.532 |
| No | 531 | 498 (93.8) | 33 (6.2) |  |  | 527 | 496 (94.1) | 31 (5.9) |  |  |
| Antiplatelet therapy |  |  |  |  |  |  |  |  |  |  |
| Yes | 251 | 233 (92.8) | 18 (7.2) | 1.290 | 0.415 | 256 | 236 (92.2) | 20 (7.8) | 1.500 | 0.196 |
| No | 416 | 393 (94.5) | 23 (5.5) |  |  | 411 | 390 (94.9) | 21 (5.1) |  |  |
| Anticoagulation therapy |  |  |  |  |  |  |  |  |  |  |
| Yes | 22 | 20 (90.9) | 2 (9.1) | 1.630 | 0.503 | 23 | 20 (87.0) | 3 (13.0) | 2.060 | 0.228 |
| No | 645 | 606 (94.0) | 39 (6.0) |  |  | 644 | 606 (94.1) | 38 (5.9) |  |  |
| Dexamethasone |  |  |  |  |  |  |  |  |  |  |
| Yes | 492 | 455 (92.5) | 37 (7.5) | 4.722 | 0.003 | 510 | 470 (92.2) | 40 (7.8) | 16.592 | 0.006 |
| No | 175 | 171 (97.7) | 4 (2.3) |  |  | 157 | 156 (99.4) | 1 (0.6) |  |  |
| Bortezomib |  |  |  |  |  |  |  |  |  |  |
| Yes | 207 | 194 (93.7) | 13 (6.3) | 1.339 | 0.394 | 196 | 181 (92.3) | 15 (7.7) | 1.647 | 0.126 |
| No | 460 | 432 (93.9) | 28 (6.1) |  |  | 471 | 445 (94.5) | 26 (5.5) |  |  |
| Cyclophosphamide |  |  |  |  |  |  |  |  |  |  |
| Yes | 126 | 116 (92.1) | 10 (7.9) | 1.453 | 0.304 | 119 | 110 (92.4) | 9 (7.6) | 1.235 | 0.577 |
| No | 541 | 510 (94.3) | 31 (5.7) |  |  | 548 | 516 (94.2) | 32 (5.8) |  |  |
| Doxorubicin |  |  |  |  |  |  |  |  |  |  |
| Yes | 111 | 100 (90.1) | 11 (9.9) | 2.197 | 0.026 | 98 | 88 (89.8) | 10 (10.2) | 2.137 | 0.037 |
| No | 556 | 526 (94.6) | 30 (5.4) |  |  | 569 | 538 (94.6) | 31 (5.4) |  |  |
| Multiagent (≥3 drugs) |  |  |  |  |  |  |  |  |  |  |
| Yes | 341 | 312 (91.5) | 29 (8.5) | 3.049 | 0.001 | 332 | 303 (91.3) | 29 (8.7) | 2.899 | 0.002 |
| No | 326 | 314 (96.3) | 12 (3.7) |  |  | 335 | 323 (96.4) | 12 (3.6) |  |  |
| Renal |  |  |  |  |  |  |  |  |  |  |
| No | 588 | 553 (94.0) | 35 (6.0) | 0.582 | 0.224 | 577 | 539 (93.4) | 38 (6.6) | 1.443 | 0.541 |
| Yes | 79 | 73 (92.4) | 6 (7.6) |  |  | 90 | 87 (96.7) | 3 (3.3) |  |  |
| Trauma |  |  |  |  |  |  |  |  |  |  |
| No | 650 | 610 (93.8) | 40 (6.2) | 1.019 | 0.985 | 648 | 608 (93.8) | 40 (6.2) | 1.280 | 0.807 |
| Yes | 17 | 16 (94.1) | 1 (5.9) |  |  | 19 | 18 (94.7) | 1 (5.3) |  |  |
| PT (s) |  | 12.68±2.34 | 12.67±2.25 |  | 0.980 |  | 12.56±2.07 | 12.87±3.18 |  | 0.417 |
| APTT (s) |  | 29.67±8.87 | 28.76±6.91 |  | 0.541 |  | 29.61±8.04 | 26.98±5.49 |  | 0.061 |
| FIB (g/L) |  | 3.14±1.02 | 3.16±1.30 |  | 0.927 |  | 3.22±1.12 | 2.83±1.08 |  | 0.052 |
| Dimer (mg/L FEU) |  | 1.73±3.44 | 3.82±7.31 |  | 0.130 |  | 1.48±2.44 | 6.09±13.88 |  | 0.079 |
| WBC (*10^9^/L) |  | 5.20±2.37 | 5.77±2.12 |  | 0.142 |  | 5.30±2.92 | 4.82±1.73 |  | 0.331 |
| Hb (g/L) |  | 99.21±24.94 | 103.87±21.96 |  | 0.256 |  | 99.35±26.62 | 98.50±20.13 |  | 0.812 |
| PLT (*10^9^/L) |  | 180.70±105.99 | 183.56±75.22 |  | 0.869 |  | 184.32±102.96 | 165.03±74.41 |  | 0.264 |
| Cr (μmol/L) |  | 105.96±104.10 | 146.49±202.47 |  | 0.223 |  | 114.94±120.40 | 110.33±151.73 |  | 0.834 |
| ALB (g/L) |  | 35.78±6.73 | 35.12±4.93 |  | 0.443 |  | 35.81±6.56 | 35.17±4.38 |  | 0.430 |
| LDH (U/L) |  | 202.12±102.74 | 183.51±51.06 |  | 0.276 |  | 188.63±69.27 | 186.68±55.32 |  | 0.873 |
| BM2G (μg/L) |  | 5329.82±5986.92 | 7397.32±9881.49 |  | 0.217 |  | 5576.77±5335.11 | 4903.16±4260.17 |  | 0.465 |
| LDL (mmol/L) |  | 2.53±1.39 | 2.68±1.91 |  | 0.591 |  | 2.55±1.38 | 2.41±0.79 |  | 0.383 |
| TG (mmol/L) |  | 2.29±2.39 | 2.01±0.97 |  | 0.185 |  | 2.23±2.35 | 2.11±1.88 |  | 0.788 |
| CHOL (mmol/L) |  | 4.46±2.16 | 3.86±1.32 |  | 0.133 |  | 12.56±2.07 | 4.41±1.02 |  | 0.892 |
