# Supplementary figures and images for: Development and validation of a new risk assessment model for immunomodulatory drug-associated venous thrombosis among Chinese patients with multiple myeloma
Source: Thromb J. 2023 Oct 4;21:105. doi: 10.1186/s12959-023-00534-y (PMC10552366; doi:10.1186/s12959-023-00534-y)

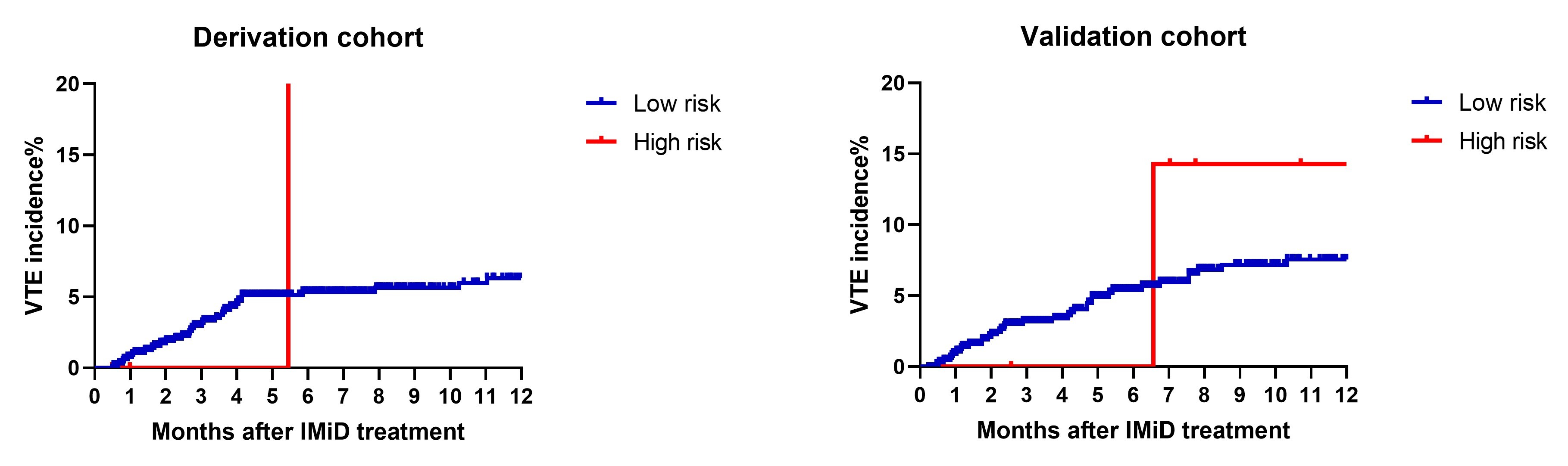

Supplement: Supplementary file 2 — Supplementary Material 2 [file 12959_2023_534_MOESM2_ESM.jpg]
